# Les modes de révélation du diabète sucré au CHU Sylvanus Olympio de Lomé

**DOI:** 10.11604/pamj.2019.34.99.20012

**Published:** 2019-01-18

**Authors:** Komi Dzidzonu Nemi, Lihanimpo Djalogue, Kodjo Agbeko Djagadou, Toyi Tchamdja, Yawovi Mawufemo Tsevi, Abago Balaka

**Affiliations:** 1 Service de Médecine Interne, CHU Sylvanus Olympio de Lomé, Université de Lomé, Togo; 2 Service de Médecine Interne, CHU Kara, Université de Kara, Togo; 3 Service de Néphrologie, CHU Sylvanus Olympio de Lomé, Université de Lomé, Togo

**Keywords:** Diabète méconnu, complications, Lomé, Togo, Unrecognized diabetes, complications, Lomé (Togo)

## Abstract

Le but de cette étude était de décrire les différentes circonstances de découverte du diabète sucré au Service de Médecine Interne du CHU Sylvanus Olympio (SO) de Lomé. Il s'agit d'une étude rétrospective menée de janvier 2015 à décembre 2017 dans le Service de Médecine Interne du CHU SO. Durant la période d'étude, 307 patients ayant le diabète sucré ont été reçu dans le service dont 104 ne se connaissaient pas diabétiques. L'âge moyen était de 51,27 ans (extrêmes de 20 et 90 ans) et la sex-ratio (H/F) de 0,5. Le diabète était de type 2 dans 75% des cas, de type 1(23%) et secondaire (2%). La découverte du diabète été fortuite (11,53%, N=12). Il a été révélé par les symptômes (26,92%, N=28) et par les complications (61,53%, N=64). Les complications étaient: Acidocétose (N=34), Syndrome d'hyperglycémie-hyperosmolaire (N=3), Hypertension artérielle (N=8), Accident vasculaire cérébral ischémique (N=4), Pied diabétique (N=2), Néphropathie diabétique (N=2), Polyneuropathie (N=1), Infections (N=18 dont 8 sont associées aux complications métaboliques. Cette étude montre que les complications constituent le mode de révélation le plus fréquent du diabète sucré dans le Service de Médecine Interne du CHU SO de Lomé. Il faut sensibiliser davantage nos populations sur la prévention et le dépistage de cette maladie.

## Abstract (en)

The purpose of this study was to describe the different circumstances in which diabetes mellitus was detected in the Department of Internal Medicine at the Sylvanus Olympio University Hospital in Lomé. We conducted a retrospective and descriptive study in the Department of Internal Medicine, at the Sylvanus Olympio University Hospital in Lomé from January 2015 to December 2017. During the study period, 307 patients with diabetes mellitus were admitted to our Department, of whom 104 did not know to have diabetes. The average age of patients was 51.27 years (ranging from 20 to 90 years), the sex ratio (M/F) was 0,5. Type 2 diabetes was diagnosed in 75% of cases, type 1 in 23% of cases and secondary in 2% of cases. The discovery of diabetes was fortuitous in 11.53% of cases (n=12). Some patients had symptoms (26.92%, n=28) while others had complications (61.53%, n=64). Complications included: ketoacidosis (n=34), hyperosmolar hyperglycemic syndrome (n=3), high blood pressure (n=8), ischemic stroke (n=4), diabetic foot (n=2), diabetic nephropathy (n=2), polyneuropathy (n=1), infections (n=18, 8 with metabolic complications). This study shows that in the Department of Internal Medicine, at the Sylvanus Olympio University Hospital in Lomé, diabetes mellitus is most commonly diagnosed when patients have complications. It is essential to educate our populations about prevention and screening tests.

## Introduction

Le diabète sucré est un problème de santé publique dans le monde et particulièrement en Afrique subsaharienne, où la constante croissance de sa prévalence constitue une préoccupation majeure [1]. Dans ces pays, cette expansion s'inscrit dans une véritable transition épidémiologique des maladies transmissibles vers les maladies non transmissibles en raison non seulement du vieillissement de la population, de la sédentarité et de l'obésité [2], mais aussi des perturbateurs endocriniens [3]. Les circonstances de découverte de la maladie varient d´un pays à l´autre en fonction du degré de médicalisation et du niveau d´intérêt des populations aux problèmes de santé [4], et sont dominées par les symptômes classiques [4-6]. Au Togo, nous ne disposons pas à notre connaissance de données hospitalières à Lomé sur les différents modes de révélation de la maladie. Le but de ce travail était de décrire les différentes circonstances de découverte du diabète sucré au Service de Médecine Interne du CHU Sylvanus Olympio de Lomé (Togo).

## Méthodes

Il s'agit d'une étude rétrospective qui s'est déroulée dans le Service de Médecine Interne (Médecine B) du CHU Sylvanus Olympio de Lomé. Elle s'est étendue sur une durée de trois ans allant du 1^er^ janvier 2015 au 31 décembre 2017. Ont été inclus dans cette étude, tous les patients non diabétiques connus avant leur admission dans le service. Le diagnostic de diabète a été posé sur la base d'une glycémie à jeun supérieure ou égale à 1,26g/l à deux ou trois reprises, ou d'une glycémie à n'importe quel moment de la journée supérieure ou égale à 2g/l à deux ou trois reprises ou encore d'une hémoglobine glyquée supérieure à 6,5%. En l'absence d'immunologie (dosage des anticorps anti glutamate décarboxylase et des anticorps anti ilots de Langerhans) et du dosage du peptide C, le typage du diabète (type 1 et type 2) a été probabiliste basé sur les arguments épidémiologiques (âge) et cliniques (morphotype et évolution clinique). Les paramètres de l'étude étaient: les données épidémiologiques (âge, sexe, type de diabète), et les circonstances de découverte du diabète.

## Résultats

**Épidémiologie:** au cours de notre période d'étude, 307 patients diabétiques ont été reçu dans le service. Parmi eux 104 ne se connaissaient pas diabétiques, soit une fréquence de 33,87% de diabète méconnu. L'âge moyen était de 51,27 ans (extrêmes : 20 et 90 ans). La tranche d'âge de 30 à 39 ans était la plus représentée (25%) suivie de celle de 50 à 59 ans (23,07%): Tableau 1. Le sex-ratio (H/F) était de 0,5 (35 hommes et 69 femmes). Le diabète de type 2 représentait 75% (N=78), le type 1 23% (N=24) et le diabète secondaire à une corticothérapie 2% (N=2).

**Tableau 1:** Répartition des patients en fonction de l’âge

Age (Année)	Effectif	Pourcentage
20-29	9	8,65
30-39	26	25
40-49	15	14,42
50-59	24	23,07
60-64	7	6,73
65-69	8	7,69
70-79	8	7,69
80-89	6	5,76
90 et plus	1	0,96
**Total**	104	100

**Circonstances de découverte:** la découverte a été fortuite chez 12 patients (11,53%) (Tableau 2). Les symptômes ont révélé le diabète chez 28 patients (26,92%): il s'agit de l'asthénie (10 cas), amaigrissement (6 cas), polyurie (20 cas) et polydipsie (20 cas) : ces symptômes pouvant être associé (Tableau 3). Les complications ont été à l'origine de la découverte du diabète chez 64 patients (61,53%). Les complications métaboliques étaient : acidocétose (N=34 ; 32,7%), Syndrome d'hyperglycémie-hyperosmolaire (N=3 ; 2,88%). Les complications chroniques macroangiopathiques étaient : Accident vasculaire cérébral ischémique (4 cas), Hypertension artérielle (8 cas), Pied diabétique (2 cas). La microangiopathie était représentée par la néphropathie au stade d'insuffisance rénale chronique (2 cas) et la polyneuropathie (1 cas). Les complications infectieuses étaient au nombre de 18 dont 8 étaient associées aux complications métaboliques: paludisme à plasmodium falciparum (1 cas), la tuberculose (2 cas), candidose vaginale (2 cas), Infection pulmonaire à germe banal (5 cas), amygdalite aigue (1 cas), otite moyenne aigue (1 cas), Erysipèle de jambe (1 cas), intertrigo inter-orteil (1 cas), infection urinaire à Escherichia coli (4 cas). La glycémie moyenne au diagnostic était de 4,09g/l (extrêmes: 1,85 et 7,2g/l) et l'hémoglobine glyquée moyenne était de 11,03% (extrêmes de 7,5 et 14).

**Tableau 2:** Répartition des patients en fonction de la découverte fortuite du diabète

Découverte fortuite	Effectif	Pourcentage
Bilan préopératoire	6	50
Douleur abdominale	2	16,66
Bilan de santé	1	8,33
Hémorroïde	1	8,33
Hémorragie digestive	1	8,33
**Total**	12	100

**Tableau 3:** Répartition des patients en fonction de l’association des symptômes ayant révélé le diabète

Symptômes	Effectif	Pourcentage
Asthénie	7	25
Asthénie+amaigrissement	1	3,57
Asthénie+polyurie+polydipsie	2	7,14
Polyurie+polydipsie+amaigrissement	5	17,85
Polyurie+polydipsie	13	46,42
**Total**	28	100

## Discussion

**Aspects épidémiologiques:** la fréquence du diabète méconnu dans notre service était de 33,87%. Cette fréquence est supérieure à celle de Monteiro *et al.* au Benin (10,7%) [4] et inférieure à celle d'Amoah *et al.* au Ghana (69,2%) [5]. Plusieurs facteurs peuvent expliquer cette variation de fréquence notamment l'intérêt particulier porté par un service à la recherche du diabète. Notre âge moyen était de 51,27 ans. La maladie a donc frappé plus dans notre série la population active dévolue à la production. Ce même constat a été fait au Benin [4] et en RDC [6]. Ceci peut se comprendre car nos travaux ont concerné des sujets âgés de 16 ans et plus. La régression des cas de diabète après 60 ans pourrait s'expliquer par la faible espérance de vie dans nos pays. La prédominance féminine retrouvée dans notre travail a été également rapportée dans une série Algérienne [7] et Rwandaise [8]. Mais certains auteurs estiment que la maladie se répartit de manière quasi-égale entre les deux genres [4-6,9]. Cette prédominance féminine dans notre série serait due à la majorité de la gente féminine dans la population Togolaise ou tout simplement à un hasard. La classification des diabétiques que nous avons adoptée était approximative. En effet, en l'absence d'immunologie et du dosage du peptide C, le typage du diabète était probabiliste basé sur les arguments épidémiologiques, cliniques et évolutifs. Malgré ces insuffisances, notre classification rejoint celle de la littérature selon laquelle le diabète de type 2 est prédominant [10-12].

**Modes de révélation:** la découverte du diabète a été fortuite chez 12 de nos patients (11,53%) et surtout dans le cadre d'un bilan préopératoire chez la moitié. Ceci montre encore une fois non seulement l'importance d'un bilan pré-thérapeutique en général qui peut permettre d'identifier une pathologie méconnue par le patient, mais aussi l'intérêt d'un dépistage volontaire ou d'un bilan annuel de santé. Les symptômes classiques ont conduit au diagnostic dans 27% des cas dans notre série contre 46% dans la série de Monteiro *et al.* au Benin [4] et 40% dans celle de Kandjingu *et al.* en République Démocratique du Congo [6]. Notre faible taux pourrait s'expliquer non seulement par la négligence de nos populations face aux problèmes de santé, mais aussi par le rôle néfaste de la médecine traditionnelle. En effet du fait des difficultés d'accès aux structures sanitaires d'une part, et des croyances religieuses d'autre part, beaucoup de malades ont recours exclusivement ou partiellement à la médecine traditionnelle qui prétend guérir de façon définitive certaines maladies chroniques [2]. Et ce n'est qu'en cas de complication que la majorité des patients sont admis dans nos services. Ainsi les complications ont été à l'origine de la découverte du diabète dans 61,53% des cas dans notre série. Les complications métaboliques étaient le mode de révélation le plus fréquent avec comme chef de file l'acidocétose (32,7%). Cette fréquence de l'acidocétose inaugurale se rapproche de celle de LEYE *et al.* [13] au Sénégal qui ont rapporté 38,24%. Le syndrome d'hyperglycémie-hyperosmolaire a inauguré la maladie chez 3 de nos patients (2,88%). Notre résultat rejoint celui de Djibril *et al.* [14] qui ont noté 2% dans le même hôpital que le nôtre mais en réanimation.

Parmi les complications chroniques ayant révélé le diabète dans notre série, la macroangiopathie a occupé une place importante et l'hypertension artérielle (HTA) occupait le premier rang. En effet l'HTA est l'une des complications macroangiopathiques les plus fréquentes du diabète [15,16]. Une surveillance régulière de la tension artérielle et la prévention des autres facteurs de risque cardiovasculaires chez le diabétique, de même qu'une éducation diabétique de qualité sont les seules armes pouvant éviter ou retarder la survenue de l'HTA chez ces patients. L'accident vasculaire cérébral ischémique (AVCI) venait en seconde position (3,8%) après l'HTA. Ce taux est proche de celui d'Ouédraogo *et al.* [11] et de Djibril *et al.* [14] qui ont rapporté respectivement 4,8% et 6,7% d'AVC. Le pied diabétique fermait la marche de la macroangiopathie dans notre série et avait concerné deux patients. Sa fréquence est diversement appréciée par les auteurs [17-19]. La microangiopathie révélatrice du diabète dans notre série était représentée essentiellement par la néphropathie diabétique au stade d'insuffisance rénale chronique (IRC). Glomérulopathie, la néphropathie diabétique constitue la première cause d'IRC terminale avec recours à l'épuration extra rénale ou à la transplantation [20] d'où l'intérêt de sa prévention par un dépistage précoce et un traitement à base d'un inhibiteur de l'enzyme de conversion ou d'un Antagoniste des récepteurs de l'angiotensine II. Les infections ont permis de découvrir la maladie chez 18 de nos patients mais il faut noter que 8 cas d'infections étaient associés aux complications métaboliques. Ceci justifie encore une fois le dépistage systématique du diabète devant toute infection notamment à répétition ou grave. Les sites infectieux restent les mêmes avec une répartition diversement rapportée dans la littérature [1,11,14].

## Conclusion

Malgré les sensibilisations de nos populations sur la prévention du diabète sucré d'une part, et son dépistage pour une bonne prise en charge d'autre part, les complications notamment l'acidocétose constituent le mode de révélation le plus fréquent de la maladie dans le Service de Médecine Interne du CHU SO de Lomé. Il faut insister davantage sur ces sensibilisations.

### Etat des connaissances actuelles sur le sujet

- Quatre principales circonstances amènent à découvrir un diabète sucré: il s'agit d'un dépistage volontaire, d'une découverte fortuite à l'occasion d'un bilan de santé ou préthérapeutique, des signes cliniques classiques et des complications;
- Ces circonstances de découverte varient d'un pays à l'autre mais sont dominées par les symptomes classiques.

### Contribution de notre étude à la connaissance

- Les complications constituent le mode de révélation le plus fréquent du diabète sucré dans le Service de Médecine Interne du CHU SO de Lomé;
- Ces complications sont dominées par l'acidocétose.
